# Unsupervised Class Generation to Expand Semantic Segmentation Datasets

**DOI:** 10.3390/jimaging11060172

**Published:** 2025-05-22

**Authors:** Javier Montalvo, Álvaro García-Martín, Pablo Carballeira, Juan C. SanMiguel

**Affiliations:** Video Processing and Understanding Lab, Escuela Politécnica Superior, Universidad Autónoma de Madrid, 28049 Madrid, Spain; alvaro.garcia@uam.es (Á.G.-M.); pablo.carballeira@uam.es (P.C.); juancarlos.sanmiguel@uam.es (J.C.S.)

**Keywords:** synthetic datasets, unsupervised domain adaptation, synthetic data

## Abstract

Semantic segmentation is a computer vision task where classification is performed at the pixel level. Due to this, the process of labeling images for semantic segmentation is time-consuming and expensive. To mitigate this cost there has been a surge in the use of synthetically generated data—usually created using simulators or videogames—which, in combination with domain adaptation methods, can effectively learn how to segment real data. Still, these datasets have a particular limitation: due to their closed-set nature, it is not possible to include novel classes without modifying the tool used to generate them, which is often not public. Concurrently, generative models have made remarkable progress, particularly with the introduction of diffusion models, enabling the creation of high-quality images from text prompts without additional supervision. In this work, we propose an unsupervised pipeline that leverages Stable Diffusion and Segment Anything Module to generate class examples with an associated segmentation mask, and a method to integrate generated cutouts for novel classes in semantic segmentation datasets, all with minimal user input. Our approach aims to improve the performance of unsupervised domain adaptation methods by introducing novel samples into the training data without modifications to the underlying algorithms. With our methods, we show how models can not only effectively learn how to segment novel classes, with an average performance of 51% intersection over union for novel classes, but also reduce errors for other, already existing classes, reaching a higher performance level overall.

## 1. Introduction

Semantic segmentation is a fundamental task in computer vision, as it enables spatial location and classification of elements in a given image. This process is crucial for various applications, including autonomous driving, medical imaging, and robotics. However, creating high-quality, pixel-level annotated datasets for training effective semantic segmentation models is labor-intensive and costly [1].

Researchers have increasingly turned to synthetic data as a viable alternative for training semantic segmentation models to address these challenges. The Synthia [2] and GTAV [3] datasets use a simulation tool and a videogame, respectively, to generate synthetic images with semantic segmentation labels and provide many advantages compared to real datasets, including perfect ground truth annotations, controlled environments, and the ability to generate large-scale datasets efficiently, without unaffordable costs. However, synthetic data also have their drawbacks: the inherent domain gap between synthetic and real-world images often leads to suboptimal performance when models trained on synthetic data are applied to real-world scenarios [4]. Moreover, existing synthetic datasets often suffer from limitations such as a reduced set of object classes or limited variability within certain categories compared to real-world data. These constraints can hinder the generalization capabilities of models trained on synthetic data.

Recently, the integration of natural language processing methods in computer vision tasks has enabled remarkable advancements in the field, particularly in the field of image synthesis with the introduction of methods like Stable Diffusion [5]. Building on this, some approaches use Stable Diffusion to generate synthetic data for semantic segmentation, such as DGInStyle [6], where images are guided using ground truths from synthetic datasets, but these synthesized images have some limitations when used for semantic segmentation, mainly due to noisy labels.

Exploiting these synthetic data also presents some challenges, as there is a notable performance degradation when training on synthetic data and measuring performance on real-domain datasets, and some works explore approaches to mitigating this degradation. Unsupervised domain adaptation (UDA) is a research line that focuses on performing supervised training on the synthetic domain while leveraging the unlabeled, real-domain data to obtain methods that generalize better between synthetic and real images. In particular, DAFormer [7] is considered a foundational work in UDA, introducing a transformer-based architecture with strong data augmentation and regularization strategies that significantly improve adaptation performance compared to previous approaches.

This paper introduces a novel approach for generating synthetic objects to expand existing datasets and their semantic masks by combining Stable Diffusion [5] and the Segment Anything Module (SAM) [8]. Our key contributions are as follows:

- We present a method that leverages the generative capabilities of Stable Diffusion alongside SAM’s accurate semantic masking to create high-quality synthetic objects with their corresponding segmentation masks.
- We demonstrate the practical applicability of our approach by successfully expanding existing synthetic datasets with additional classes without requiring architectural modifications to semantic segmentation methods.
- We show that models can effectively learn these newly generated classes within unsupervised domain adaptation (UDA) pipelines, achieving performance comparable to the original classes in the dataset.

### 1.1. Related Work

#### 1.1.1. Generating Synthetic Data

Synthetic datasets are a valuable tool for semantic segmentation pipelines, offering significant advantages over manually collected data. The generation of labeled synthetic imagery drastically reduces data gathering costs while enabling efficient and scalable data production. This approach also allows precise control over environmental conditions, object positioning, and edge cases that would be difficult or dangerous to capture in real-world settings. Despite these benefits, synthetic data present a fundamental challenge: models trained on synthetic imagery typically exhibit reduced performance when deployed on real-world data compared to their performance on the synthetic domain itself [4]. This performance gap remains a central obstacle that various data generation and domain adaptation techniques attempt to overcome. In the past, synthetic data were generated using synthetic environments, such as videogames or simulation tools, but recently, with the surge in generative AI, some recent works have also explored generating synthetic data through diffusion models.

Synthetic environments have been used for data gathering and testing in different tasks, as they provide a simple and effective playground that not only proves to be cost-efficient but also enables the generation of data that may be dangerous or too costly to collect.

In recent years, synthetic data have usually been generated with tools designed specifically for the task of data simulation and gathering. CARLA [9] and LGSVL [10], are widely used tools designed for simulating autonomous driving tasks; there are also tools for different purposes, such as AirSim [11] for simulating drone navigation. There are also commonly used synthetic datasets like Synthia [2] or SynLiDAR [12], generated with unpublished tools. Additionally, modified videogames can also be a useful source for synthetic data: The GTA Dataset [3] was captured using the Grand Theft Auto V video game, and although it required manual label curation, it has become a staple dataset in the field, as it leverages the variability and image quality of a multi-million dollar entertainment project to obtain useful ground-truth labels for different tasks.

Yet, creating these tools and generating these datasets have two notable limitations: (1) extensive human labor with specific knowledge is required to build these tools, and (2) they offer a limited class variety constrained by the available 3D assets.

Diffusion modelshave disrupted the field of generative artificial intelligence, as they can generate realistic imaging just from a set of text prompts, without specific tuning, and all of this while ensuring high intra- and inter-class variability. Yet, they have some limitations that restrict their usage when generating synthetic data for semantic segmentation. The extraction of pixel-accurate semantic labels from images generated with diffusion models is still an open problem, as it has to solve two particular challenges: correctly identifying the class of the depicted object, and the pixel-perfect localization of the object in the image, as the noise introduced by label misalignment can result in performance degradation on the semantic segmentation models. Some works try to exploit the capabilities of Stable Diffusion for generating synthetic images for semantic segmentation: DatasetDM [13] introduces a generation model with capabilities to generate images and their ground-truth annotations, relying on a segmentation decoder to achieve image–mask alignment. More recently, DGInStyle [6] relied on ControlNet to generate a diverse dataset of street scenes with consistent image–label alignment by guiding the generation with a semantic segmentation ground-truth image, obtaining different images that represent the same scene. But, due to the nature of diffusion methods, both alternatives show limitations, with pixel misalignments between the synthetic image and its ground truth.

#### 1.1.2. Exploiting Synthetic Data

Although using synthetic environments to generate data has many advantages, it also has some drawbacks and limitations [4]. They often have reduced variability compared to real-world images, due to a limited amount of 3D objects; for example, CARLA [9] has 18 different car models, but there are thousands in the real world. Also, these environments often lack realism, and seeking realism can increase the development costs of the tool quickly to the point where it may surpass the cost of gathering and labeling real datasets.

These differences are referred to as the domain gap, and overcoming this domain gap is one of the most important tasks in semantic segmentation, called domain adaptation. The domain adaptation task involves a source domain, where models are trained on an annotated dataset, and a target domain, where the model is intended to be deployed. Ideally, the source synthetic data should substitute real data during training, with the objective of leveraging the knowledge gained from the synthetic source domain to enhance model performance in an unlabeled real target domain.

In this work, we focus on the task of unsupervised domain adaptation (UDA), where we exploit the unlabeled data of the target domain to further enhance the performance of an algorithm trained with supervision on the source domain.

UDA tries to leverage data from both domains to increase the performance of a given algorithm on a target domain. This adaptation can be performed at different levels, and we can distinguish between three main trends for unsupervised domain adaptation depending on the space where the adaptation is performed [4]: In input-space domain adaptation [7,14,15,16,17], the adaptation is performed by modifying the images used to train the segmentation model. In feature-space domain adaptation [18,19,20,21,22], the adaptation is performed at a feature level within the model, usually by trying to align feature distributions between domains; and in output-space domain adaptation [7,23,24,25,26] the adaptation is performed by trying to align predictions between the source and target domains.

In our work, we propose including new classes in the source-domain space, without any specific knowledge of the target domain.

#### 1.1.3. Segment Anything Module

The Segment Anything Module (SAM) [8] is a breakthrough in the realm of segmentation models, designed to generalize across a wide variety of segmentation tasks without requiring extensive task-specific training. The SAM can use different types of input prompts, such as points, boxes, or masks, to produce zero-shot segmentation across diverse datasets, addressing the challenges posed by domain variability. By learning to segment “anything”, the SAM offers a robust solution that can significantly reduce the reliance on large annotated datasets, thereby accelerating the development and deployment of segmentation models across different domains. The main advantage of this model is its ability to produce high-quality semantic segmentation masks, with the drawback being that no semantic information about the segmented object is provided.

This paper is organized as follows: First, we have introduced our objective and some relevant works; we follow this in Section 2 by introducing our generation pipeline and data combination method; followed by Section 3, where we show the results of the different experiments explored to prove the applicability of our method.

## 2. Methods

**Figure 1:**
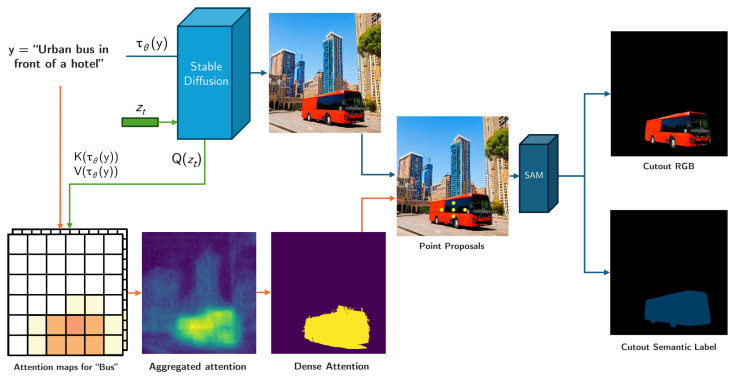
Schematic of our pipeline. The upper path contains the generation of the synthetic image and the lower path depicts the process of obtaining the semantic mask for the generated example.

We propose an unsupervised and training-free pipeline (see Figure 1) designed to generate samples of new classes so they can be included in already existing datasets.

### 2.1. Pipeline Definition

The general scheme for our class sample generation process is shown in Figure 1. First, we generate a list of text prompts ${\left\{p_{j}\right\}}_{j=0}^{N}$ by randomly mixing a list of possible class types and possible locations; e.g., for the class *bus*, we would have different class examples like *school bus, tour bus*, and *trolleybus*, among others, and then a list of locations for that class to ensure visual variability, e.g., *in the street, at the airport, on a scenic route*, etc.

These $p_{j}$ prompts are used as text conditioning for a Stable Diffusion [5] model D that, for a given noise vector $z_{t}$, generates an image $I_{j}$, defined as

(1) $$I_{j}=D\left(\epsilon_{\theta }\left(z_{t},t,\tau_{\theta }\left(p_{j}\right)\right)\right)$$

We also store the queries $Q_{i,t}$, corresponding to the spatial location *i* at time step *t*, from the denoising process. Now, we localize the object from the new class in the image using the attention for its class token. To maximize this attention, and following the method from [27], we use a modified version of our input prompt $\bar{p_{j}}$ where we simplify the class definition, trying to reduce the semantic class to a single token. For example, for our *bus* example, we would change *school bus* to *bus*. The text embedding of this modified prompt $\tau_{\theta }\left(\bar{p_{j}}\right)$ is then processed to produce the associated linear projections for its attention $\bar{K}$, defined as

(2) $$\bar{K}=W_{\bar{K}}^{\left(i\right)}\cdot \tau_{\theta }\left(\bar{p_{j}}\right)$$

This attention $\bar{K}$ is then combined with the pixel queries to create open-vocabulary attention matrices $A(Q_{i,t},{\bar{K}}_{i})$. We then resize the attention matrices to a common resolution and aggregate the matrices across layers, time steps, and attention heads, similarly to DAAM [28]:

(3) $$D_{\tau_{\theta }\left(p_{j}\right),k}\left(\tau_{\theta }\left(\bar{p_{j}}\right)\right)=\sum_{i,t,h}\left(A_{h,k}\left(Q_{i,t},\bar{K_{i}}\right)\right)\in R^{W\times H}$$

This defines the attention matrix for each *k*-th token. We then select the attention matrix for our new class token, $M_{q}$, defined as

(4) $$M_{q}=D_{\tau_{\theta }\left(p_{j}\right),Q}$$

From this matrix, we filter out the pixels with attention values below a given threshold *T*, which we set at 0.5. After this filter, we use a DenseCRF [29] to post-process the filtered attention map and obtain a dense binary mask, which is then used to obtain a bounding box that gives the location of the object from the class in the generated image **$I_{j}$**.

We use this bounding box to propose five points located in a cross pattern around the bounding box center and use them to prompt the SAM [8] to obtain the $M_{j}$ binary mask for our object, which we then apply to the generated image **$I_{j}$** to produce the RGB cutout and semantic segmentation ground truth for the new class.

Using variations of the automatically generated prompts and different latent vectors $z_{t}$, we can repeat this process multiple times to obtain a large set of RGB cutouts $x_{q}$ and their semantic masks $y_{q}$.

### 2.2. Mask Curation

**Figure 2:**
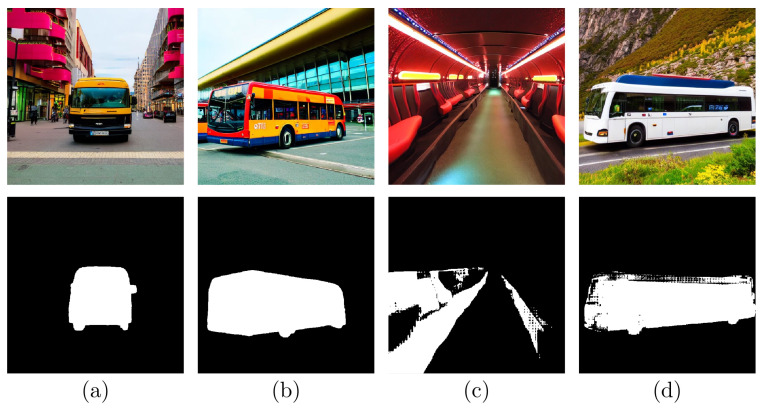
Images (**a**,**b**) show examples of valid images and masks for the *bus* class; (**c**) shows what seems to be the interior of a bus, and (**d**) has a noisy mask, so both are discarded.

Repeating this method with different latent noises and prompts, we can obtain varied cutout examples for the new class we want to include in the dataset, although they require an additional curation step, as some of them may present some issues. Figure 2 shows some examples of generated images and class masks. For example, when the attention mask occupies a notable proportion of the image, we can assume either it is a close-up, or it is highly probable that the image is not something we want (for example, image (c) in Figure 2 resembles the interior of a bus). We discard these images just by setting a fixed threshold for the ratio of attention pixels, which we set at 40% of the image.

The (d) example from Figure 2 shows one of the drawbacks of the SAM: it may produce noisy segmentation masks, particularly on object edges, as the SAM is trained on 1024 × 1024 images, and the resolution of the images we generate is 512 × 512.

To remove these samples, we include an additional filtering process, relying on three different metrics to filter these noisy masks: the Polsby–Popper method for measuring mask compactness, a mask contour smoothness metric, and measuring angular change along the mask of the contour metric.

The Polsby–Popper [30,31] method is defined as

(5) $$PP\left(m\right)=\frac{4\pi *A(m)}{P{\left(m\right)}^{2}}$$

where *m* is the mask generated by the SAM, A(m) is the area of the mask, and P(m) is the perimeter of the mask. We only keep masks where PP(m)>0.6.

Masks that pass this initial filter are then processed using a perimeter smoothness metric.

(6) $$S\left(m\right)=\frac{P(m)}{P_{s}\left(m\right)}$$

where $P_{s}\left(m\right)$ is the perimeter obtained after smoothing the mask. If S(m)<1.0, the mask is discarded.

Finally, we process the mask using an energy metric that measures the angular change along the perimeter contour, defined as

(7) $$E_{P}=\sum_{i=1}^{N-1}\Delta \theta_{i}$$

where $\theta_{i}$ is the angle between two consecutive segments in the contour, so the energy for two consecutive angles would be $\Delta \theta_{i}=\left|\theta_{i+1}-\theta_{i}\right|$. A high energy value indicates more irregular or noisy boundaries, with larger or more frequent changes in direction. We keep masks where $E_{P}<50$. Although we could normalize this energy using the perimeter length, we found this often resulted in bigger masks with smaller noisy portions not being discarded. Thresholds were selected after manual observation of a small subset of 20 images for the *bus* class and were kept the same for other classes.

### 2.3. Including Novel Classes in Unsupervised Domain Adaptation Pipelines for Semantic Segmentation

To validate our pipeline, we include our synthesized cutouts in a UDA framework. In UDA, we have two sets of images: A *source* domain $X_{S}={\left\{(x_{s}^{i},y_{s}^{i})\right\}}_{i=0}^{N_{s}}$, composed of images with ground-truth semantic labels $y_{r}^{i}\in {\left\{1,2,\ldots ,C\right\}}^{H\times W}$; and a *target* domain $X_{T}={\left\{(x_{t}^{i})\right\}}_{i=0}^{N_{t}}$, with images but not labels, to leverage the source data to train an algorithm that effectively generalizes to the target domain. Usually, this is achieved by performing supervised training on the source data, and then using a teacher model or even the same model to generate pseudo-labels for the target data [7,14,15,32], which are then used in the loss computation for target images.

To include our novel class examples, we propose a method that works by modifying the data used to train on the supervised source domain. Drawing inspiration from MixUP [16], we randomly combine source images $x_{s}^{i}$ and our class examples $x_{q}$ with a fixed probability $p_{m}$, by computing the dot product of the new class mask $y_{q}$ and the source image at a random location where the bounding box of $x_{q}$ is fully contained within the dimensions of the source image. With this, for a copy of the source image and labels $x_{m}^{i}=x_{s}^{i}$ and $y_{m}^{i}=y_{s}^{i}$, we randomly propose a region of the source image roi where the cutout can be overlaid $x_{m}^{i}\left[roi\right]$, and then substitute the pixels inside this region with the content of the new class:

(8) $$x_{m}^{i}\left[roi\right]=y_{q}^{i}\cdot x_{q}^{i}+\left(1-y_{q}^{i}\right)\cdot x_{m}^{i}\left[roi\right]$$

**Figure 3:**
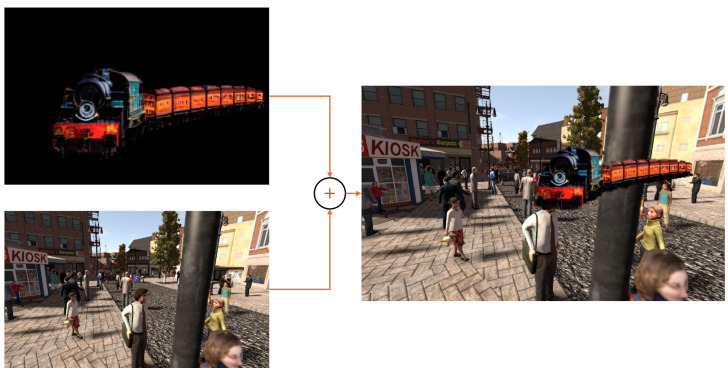
Example of our novel class sample inclusion method.

Similarly, we substitute the semantic segmentation labels with the novel class index *Q*,

(9) $$y_{m}^{i}\left[roi\right]=y_{q}^{i}\cdot Q+\left(1-y_{q}^{i}\right)\cdot y_{m}^{i}\left[roi\right]$$

obtaining new image–label pairs $x_{m}^{i},y_{m}^{i}$ with $y_{m}^{i}\in {\left\{1,2,\ldots ,C,Q\right\}}^{H\times W}$, increasing the number of classes present in the labels from the source domain. Figure 3 shows an example of the resulting training image generated with our mix approach by combining a cutout of the class *train* generated with our pipeline and an image from the Synthia [2] dataset. For simplicity and consistency with DAFormer’s [7] MixUp [16] implementation, we do not perform augmentation on the cutouts. In Figure 3, we show an example of a synthetic sample for the class train being included into an image from the Synthia dataset.

## 3. Results

### 3.1. Experimental Setup

All our tests were performed using the DAFormer [7] pipeline for UDA. All models were trained for 40,000 iterations, with a batch size of 2, using standard DAFormer settings.

We rely on Cityscapes [1] to measure our method’s performance after training on two different synthetic datasets: CARLA-4AGT [33], which contains 16 out of 19 of the Cityscapes classes, missing *bus*, *train*, and *terrain*; and the Synthia dataset [2], which contains 16 out of 19 Cityscapes classes, missing *train*, *truck*, and *terrain*.

**Figure 4:**
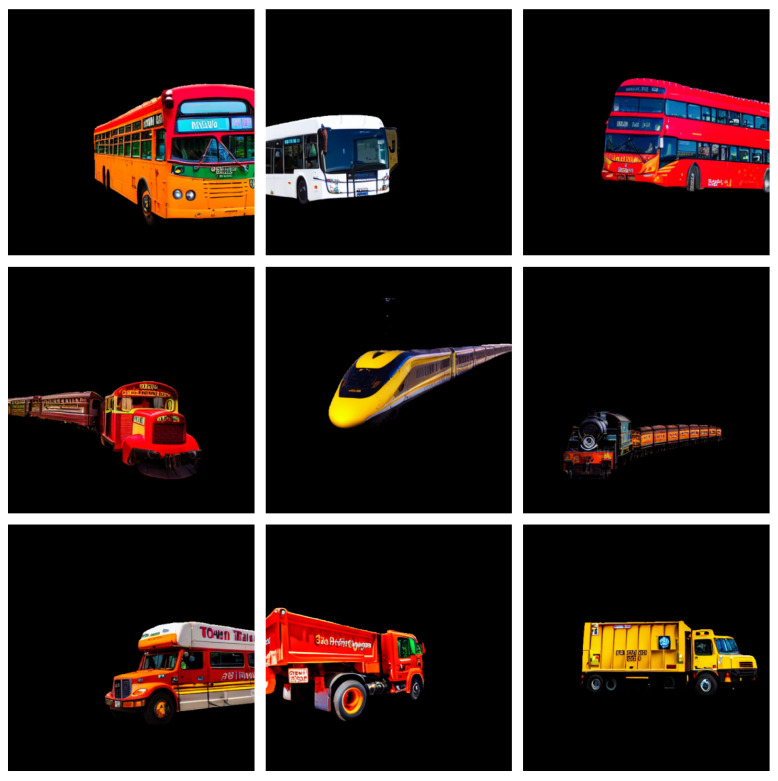
Each row shows cutout examples for *bus*, *train*, and *truck* in descending order.

For our tests, we generated samples of the *bus*, *truck*, and *train* classes. To create the cutouts for these classes we started by prompting the open-source conversational model Llama 2 [34] for a list of possible types of vehicles for each class, and another list of possible locations. Additionally, in the prompt, we included the styling words *ego camera*, and *color*, and included a negative prompt with the content *grayscale, artistic, painting* to maximize the output of images that resembled photographs. With these prompts, we generated images for all three classes, using our pipeline to extract cutouts and masks, and our filtering process. Figure 4 shows some filtered cutout examples for the different *bus*, *train*, and *truck* classes.

In all our tests, we measure performance by calculating the per-class intersection over union (IoU) [35] between the model predictions and the ground-truth labels of the Cityscapes validation set, with IoU defined as

(10) $$\mathrm{IoU}=\frac{TP}{TP+FP+FN}$$

where TP and FP represent *true-positive* and *false-positive* pixels, respectively, and FN represents the amount of false-negative pixels.

### 3.2. Including New Classes in Datasets

For this experiment, we generate 2000 cutouts for each class in *bus*, *truck*, and *train*, and include them into source images using our proposed mix method.

In Table 1, we show the results after the inclusion of the classes each dataset was missing compared to Cityscapes, both individually and at the same time. Including our cutouts is not only useful for learning a class that was previously missing from the training data, allowing these novel classes to be effectively segmented, but also results in performance benefits for other classes.

**Figure 5:**
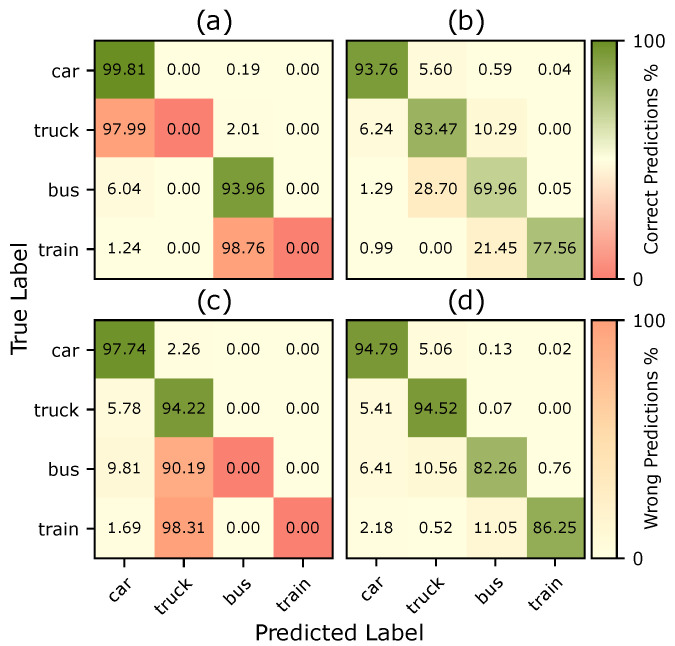
Confusion matrices for vehicle classes before and after including the two missing classes. (**a**) Synthia baseline; (**b**) Synthia + our approach; (**c**) CARLA-4AGT; (**d**) CARLA-4AGT + our approach.

This performance uplift for other classes is more evident when looking at the confusion matrices from Figure 5, where we can see how *train* elements were being predicted as *bus* and *trucks* were predicted as *cars* when adapting from Synthia to Cityscapes. Still, with the inclusion of our synthesized cutouts, the model is now able to segment the *truck* class properly. For the model trained with the CARLA-4AGT dataset, we see a similar behavior, but in this case the *bus* and *train* classes are being wrongly labeled as *truck*.

### 3.3. Ablation Tests

#### 3.3.1. Impact of Appearance Rate

**Figure 6:**
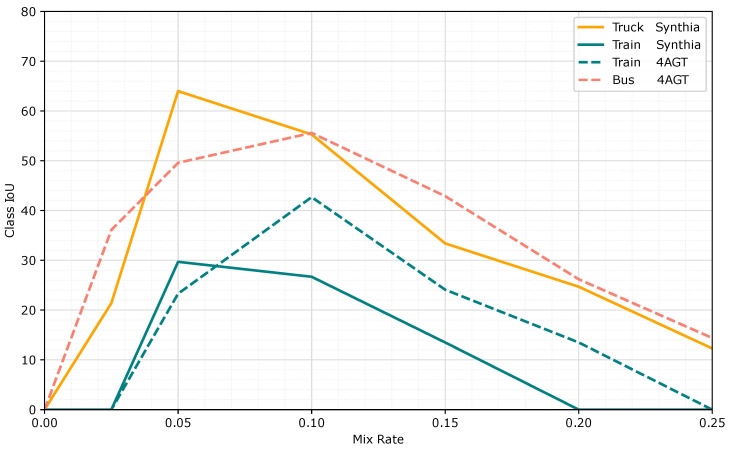
Performance of the *bus*, *truck*, and *train* classes when using our approach to the complete Synthia and CARLA-4AGT datasets. The optimal value varies across datasets.

We performed an ablation test for different values of the $p_{m}$ parameter that decides the probability of a novel class being inserted into an image. In Figure 6, we show the per-class performance of new classes depending on the mixing probability parameter. If the parameter is too high, the model overfits and loses generalization capability. If the parameter is too low, the number of examples seen is not high enough, and the model is not able to learn to segment the new class. We find that the optimal parameter value varies across datasets. We believe this is due to the differences in image sizes between Synthia (1280 × 760) and CARLA-4AGT (2048 × 1024), which results in the cutouts occupying a smaller region of the image for CARLA-4AGT images; so, when performing the 512 × 512 crop during training, there is a smaller probability of sampling the cutout, so a higher $p_{m}$ value compensates for the smaller chance of being cropped.

#### 3.3.2. Mask Filtering Evaluation

To evaluate the impact of the cutout filtering described in Section 2.2, we now train the same DAFormer setup using 2000 unfiltered cutouts for the *bus*, *truck*, and *train* classes and compare them to the results after training on 2000 filtered images. The results are summarized in Table 2. For all classes, there is a performance degradation when not using mask filtering, and we can see how some classes seem to be slightly affected by not filtering cutouts (Synthia+Truck), while other classes suffer a noticeable drop in performance, also affecting the overall model performance; for example, when training Synthia including unfiltered train cutouts, we observe a global mIoU performance drop compared to the baselines (53.8 vs. 54.9). Novel-class performance degradation when using unfiltered cutouts is aligned with the amount of generated samples required to obtain the 2000 valid cutouts after filtering: For the *truck* class, we had to generate 2600 images and masks to reach 2000 *valid* cutouts, while the *train* class, that showed more performance degradation, required 3700 generations. This suggests the filtering process is necessary to discard samples that hinder the performance of models when training with them.

## 4. Discussion

In this work, we present an automatic pipeline that leverages Stable Diffusion to generate synthetic class examples that can be used to train semantic segmentation algorithms, and propose a method for exploiting this pipeline by including novel classes in existing semantic segmentation datasets to extend their semantic categories. We show results in unsupervised domain adaptation pipelines that prove that our method successfully enables learning classes not originally available in the source datasets with a performance similar to that obtained for other existing classes.

### Future Work

Our pipeline opens several directions for future research:

- Applying the proposed pipeline as an adversarial sample generator for hard classes where segmentation models perform poorly to improve model robustness.
- Extending the method to enable unsupervised generation of complete synthetic datasets by composing multiple class instances into randomized scenes, following the principles of domain randomization [36].
- Measuring how this technique can help with rare or under-represented classes by creating new samples to increase their intra-class variability and address class imbalance in long-tail distributions.

## 5. Conclusions

This paper introduces a novel approach for extending semantic segmentation capabilities through synthetic data generation. Our method exploits attention maps from Stable Diffusion combined with the Segment Anything Module to automatically create and filter synthetic class samples. By incorporating these samples into existing datasets through a MixUp-inspired approach, we enable training for novel classes within the DAFormer pipeline for unsupervised domain adaptation. The results demonstrate that models can learn to segment new classes using only our synthetic examples, achieving an intersection over union comparable to established classes in existing datasets. The approach reduces segmentation error for the original classes while requiring minimal human supervision, providing an efficient method for dataset augmentation and class extension in semantic segmentation tasks. Future efforts can build on this work to develop fully synthetic training pipelines, improve model robustness to challenging classes, and address long-tail class distributions in a scalable manner.

## Figures and Tables

**Table 1:** Per-class performance in the DAFormer UDA pipeline. Classes introduced with our approach are in italics. Best-performing model per class is shown in bold. “–” indicates the class is not present in the dataset. (Synthetic → Cityscapes).

	New Class	Road	Sidewalk	Building	Wall	Fence	Pole	Traffic Light	Traffic Sign	Vegetation	Sky	Person	Rider	Car	*Truck*	*Bus*	*Train*	Motorcycle	Bicycle	mIoU
Synthia	-	82.4	37.7	**88.7**	43.0	**8.4**	**50.8**	**55.7**	55.1	86.0	88.1	74.2	**49.5**	87.8	-	63.2	-	54.5	**62.8**	54.9
	Train	**87.5**	**50.2**	88.4	44.4	1.6	49.2	53.1	50.7	85.3	92.8	**74.5**	48.2	85.9	-	**70.6**	*29.7*	53.4	60.1	57.0
	Truck	82.5	40.8	88.8	**44.6**	6.8	50.4	55.5	51.0	85.1	91.7	67.1	47.6	**90.5**	***64.0***	60.3	-	**55.8**	62.2	58.0
	Both	86.5	47.1	88.3	44.4	4.4	49.9	54.1	**54.8**	**86.6**	**93.0**	73.1	41.2	86.3	*38.4*	49.2	***52.0***	53.5	60.8	**59.1**
4AGT	-	91.5	68.5	89.1	43.4	30.1	50.1	48.4	59.8	88.3	92.7	70.7	35.4	88.0	25.7	-	-	49.9	**61.9**	55.2
	Train	**96.1**	70.1	88.9	44.4	29.9	50.2	54.3	**62.3**	88.1	92.7	69.9	41.9	86.9	**66.1**	-	*42.7*	54.8	58.4	61.0
	Bus	96.0	70.7	**89.2**	**44.6**	**33.8**	**51.8**	**54.4**	60.6	**88.6**	**93.7**	69.6	38.6	**90.1**	56.7	*49.5*	-	52.0	60.3	61.1
	Both	95.9	**70.5**	87.5	33.7	25.9	51.0	53.0	57.6	88.3	93.2	**70.4**	**43.1**	85.5	35.4	**65.2**	***65.5***	***55.1***	61.0	**63.2**

**Table 2:** Performance comparison when training with and without mask filtering. Novel classes in italics. Best-performing model per class is shown in bold. “–” indicates the class is not present in the dataset. (Synthetic → Cityscapes).

	Class	Filtering	Road	Sidewalk	Building	Wall	Fence	Pole	Traffic Light	Traffic Sign	Vegetation	Sky	Person	Rider	Car	*Truck*	*Bus*	*Train*	Motorcycle	Bicycle	mIoU
Synthia	Train	✗	85.9	45.6	88.5	45.7	8.3	50.1	54.3	48.8	86.8	90.6	73.1	40.2	89.4	-	57.3	*0.0*	49	54.3	53.8
		✓	87.5	50.2	88.4	44.4	1.6	49.2	53.1	50.7	85.3	92.8	74.5	48.2	85.9	-	70.6	***29.7***	53.4	60.1	57.0
	Truck	✗	85.4	43.5	89.1	47.4	9.2	49.9	54.5	56.1	86.4	87.9	69.0	43.1	88.9	*61.2*	51.2	-	53.2	61.3	57.6
		✓	82.5	40.8	88.8	44.6	6.8	50.4	55.5	51.0	85.1	91.7	67.1	47.6	90.5	***64.0***	60.3	-	55.8	62.2	58.0
4AGT	Train	✗	96.1	64.5	88.8	41.2	27.5	50.2	53.1	59.8	88.2	92.8	69.9	41.9	88.3	26.4	-	*13.2*	51.2	62.1	56.4
		✓	96.1	70.1	88.9	44.4	29.9	50.2	54.3	62.3	88.1	92.7	69.9	41.9	86.9	66.1	-	***42.7***	54.8	58.4	61.0
	Bus	✗	95.4	70.3	88.7	44.2	33.2	52.3	52.1	61.3	88	93.4	68.3	44.4	87.5	47.0	*28.3*	-	49.1	63.2	59.3
		✓	96.0	70.7	89.2	44.6	33.8	51.8	54.4	60.6	88.6	93.7	69.6	38.6	90.1	56.7	***49.5***	-	52.0	60.3	61.1

## Data Availability

We will share the code used to generate and curate our synthetic datasets, and the code used to include the novel classes in existing datasets upon acceptance at the repository: https://github.com/vpulab/unsupervised-class-generation (accessed on 19 May 2025).

## Abbreviations

The following abbreviations are used in this manuscript:

UDA	Unsupervised domain adaptation
SAM	Segment Anything Module
